# HARP Versus SinMod for measuring regional heart function from tagged CMR images

**DOI:** 10.1186/1532-429X-18-S1-P60

**Published:** 2016-01-27

**Authors:** El-Sayed H Ibrahim, Scott Swanson, Jadranka Stojanovska, Claire Duvernoy, Rodica Pop-Busui

**Affiliations:** grid.214458.e0000000086837370University of Michigan, Ann Arbor, MI

## Background

CMR tagging is a valuable technique for evaluating regional heart function. Currently, there are a number of different techniques for analyzing the tagged images, which are based on different analysis algorithms. The purpose of this study is to compare the harmonic phase (HARP) and sine-wave modeling (SinMod) tagging analysis techniques for evaluating myocardial strain and torsion in healthy controls (HC) and patients with type-1 diabetes (T1DM).

## Methods

**Figure 1:**
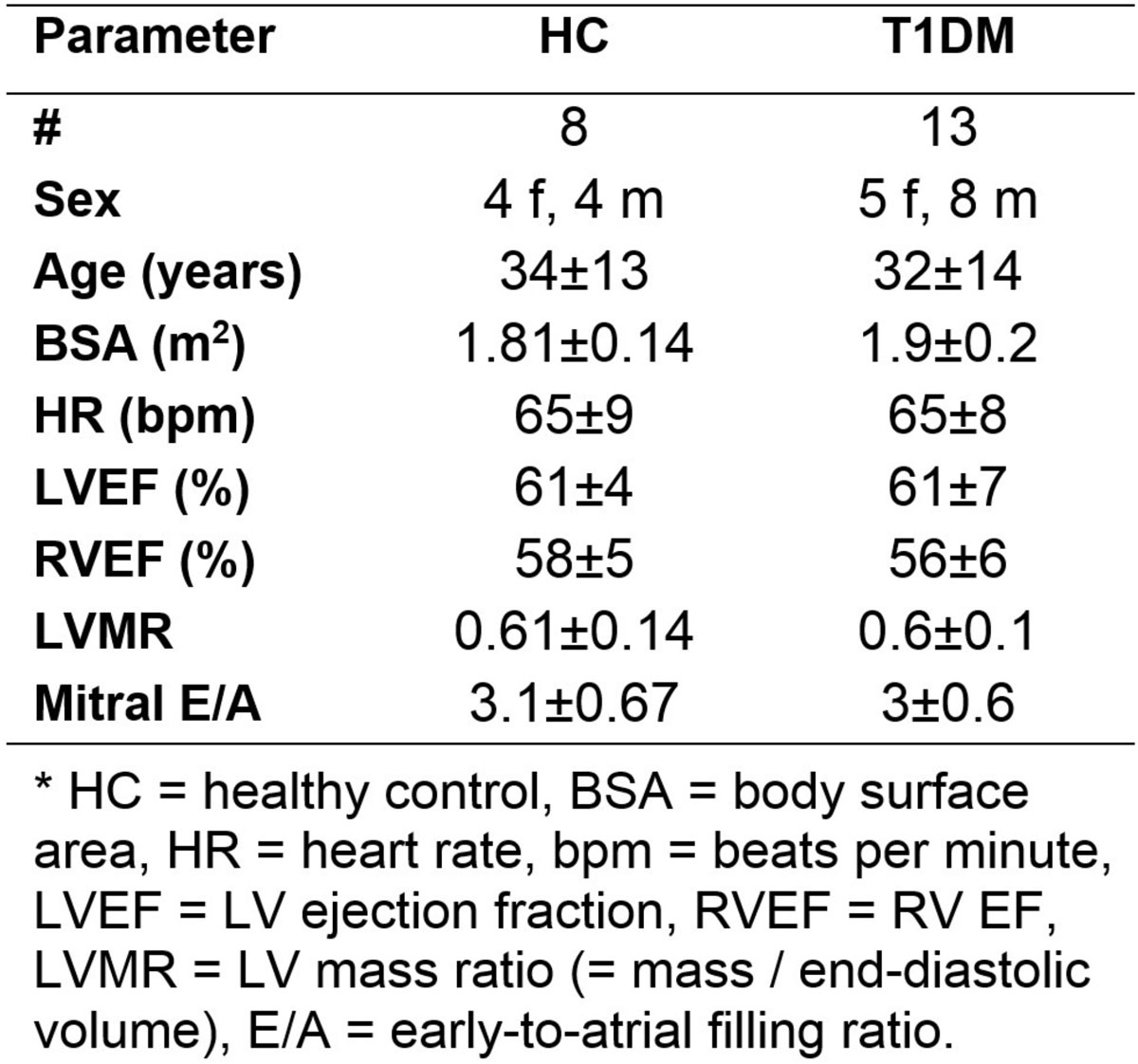
**Subjects' characteristics**.

13 T1DM patients and 8 matched HC (Figure 1) underwent CMR exam that included cine, tagged, and mitral flow images, which were analyzed to measure ventricular mass and ejection fraction (EF), strain and torsion, and mitral early-to-atrial flow rate (E/A), respectively. The tagged images were analyzed by an expert using HARP by Diagnosoft and SinMod by InTag. The tagging analysis was conducted on short-axis slices at the base, mid-ventricular, and apical levels to measure circumferential strain and apical-to-base torsion. Correlation analysis was conducted to evaluate the relationship between HARP and SinMod measurements. Student's t-test was conducted to evaluate the significance of the measurements' differences between HARP and SinMod as well as between patients and HC (P < 0.001 was considered significant).

## Results

**Figure 2:**
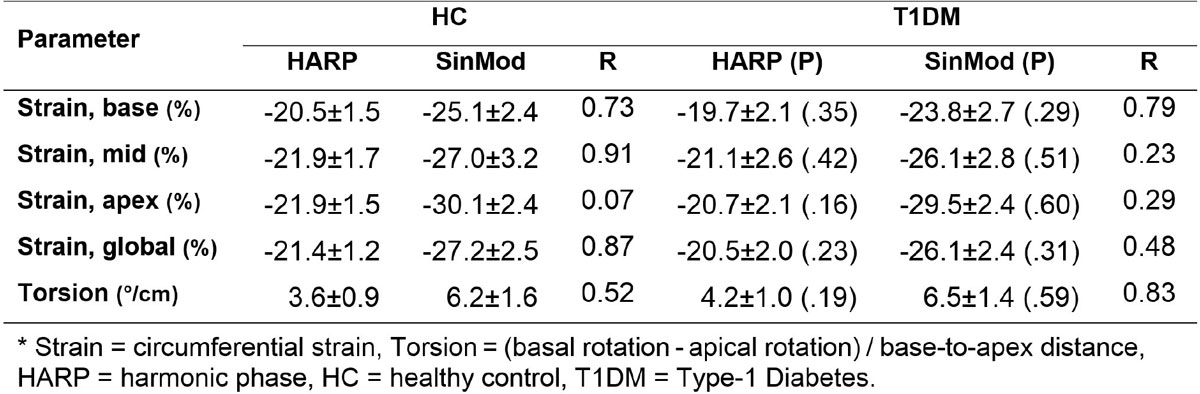
**Myocardial strain and torsion by HARP and SinMod in HC and T1DM patients**.

As shown in Figure 2, all SinMod measurements were significantly larger than those by HARP. Nevertheless, there existed consistency in the measurements by each technique, as seen by the good correlation between the HARP and SinMod measurements in both normals and patients, except in apical strain (both groups) and mid strain in patients. Further, the two techniques resulted in close P values between the measurements in patients and HC, except for apical strain and torsion. For all measurements, P was insignificant between patients and HC.

## Conclusions

The analysis algorithms in HARP and SinMod are significantly different. While HARP is based on analyzing the signal phase of the extracted harmonic peaks in the tagged images, SinMod is based on modeling the intensity distribution in the surrounding of each pixel as a summation of sine wavefronts. The results clearly showed measurements' exaggeration by SinMod compared to HARP. Nevertheless, there existed consistency in the measurements by each technique, as described in the Results section. Most of the discrepancy between the two techniques occurred in apical measurements, which can be attributed to the small anatomical size (i.e. fewer taglines) and lower image quality in the apical images. The insignificant differences between patients and HC is not unexpected in T1DM due to the young age of the patients and nature of disease progression, e.g. compared to Type-2 diabetes. In conclusion, care should be taken no to mix measurements from different tagging analysis techniques. Further studies including ground-truth measurements are needed to compare the performance of different tagging analysis techniques.

